# Synthesis and Characterization of Gd-Functionalized B_4_C Nanoparticles for BNCT Applications

**DOI:** 10.3390/life13020429

**Published:** 2023-02-02

**Authors:** Agostina Vitali, Maria Paola Demichelis, Greta Di Martino, Ian Postuma, Silva Bortolussi, Andrea Falqui, Chiara Milanese, Chiara Ferrara, Patrizia Sommi, Umberto Anselmi-Tamburini

**Affiliations:** 1 Department of Chemistry, University of Pavia, V.le Taramelli 12, 27100 Pavia, Italy; 2 Unit of Pavia, National Institute of Nuclear Physics, INFN, Via A.Bassi 6, 27100 Pavia, Italy; 3 Department of Physics, University of Pavia, Via A.Bassi 6, 27100 Pavia, Italy; 4 Department of Physics “Aldo Pontremoli”, University of Milan (Statale), Via Celoria 16, 20133 Milan, Italy; 5 Department of Material Science, University of Milano Bicocca, Via R. Cozzi 55, 20125 Milan, Italy; 6 Department of Molecular Medicine, University of Pavia, Via Forlanini, 27100 Pavia, Italy

**Keywords:** BNCT, boron carbide nanoparticles, gadolinium nanoparticles

## Abstract

Inorganic nanoparticles of boron-rich compounds represent an attractive alternative to boron-containing molecules, such as boronophenylalanine or boranes, for BNCT applications. This work describes the synthesis and biological activity of multifunctional boron carbide nanoparticles stabilized with polyacrylic acid (PAA) and a gadolinium (*Gd*)-rich solid phase. A fluorophore (DiI) was included in the PAA functionalization, allowing the confocal microscopy imaging of the nanoparticles. Analysis of the interaction and activity of these fluorescent *Gd*-containing *B*_4_*C* nanoparticles (FGdBNPs) with cultured cells was appraised using an innovative correlative microscopy approach combining intracellular neutron autoradiography, confocal, and SEM imaging. This new approach allows visualizing the cells, the FGdBNP, and the events deriving from the nuclear process in the same image. Quantification of ^10^*B* by neutron autoradiography in cells treated with FGdBNPs confirmed a significant accumulation of NPs with low levels of cellular toxicity. These results suggest that these NPs might represent a valuable tool for achieving a high boron concentration in tumoral cells.

## 1. Introduction

Boron neutron capture therapy (BNCT) represents a promising binary radiation therapy for difficult-to-treat tumors. It takes advantage of the high propensity of the nonradioactive isotope ^10^*B* to adsorb a neutron to form an excited ^11^*B* nucleus, decaying immediately and emitting an alpha particle, a ^4^*He*^2+^ ion, and a ^7^*Li*^3*+*^ ion. The energy released in this reaction is approximately 2.8 MeV. The decay products present a high linear energy transfer (LET) and a total range in the tissue of about 14 µm, a distance comparable with the dimension of a single cell. As a result, the decay products can selectively destroy cells that have taken up enough ^10^*B* while sparing the others [1,2,3]. Hence, its use in tumor therapy is associated with the possibility of selectively enriching the tumor tissues with ^10^*B*.

To deliver a therapeutic dose to the tumor in a reasonable irradiation time with the available neutron beams, ^10^*B* concentrations of tens of ppm are necessary. At the same time, to avoid damage to the normal tissues, a concentration as low as possible is required in healthy cells. Hence, for the BNCT to be successful, a highly targeted delivery of ^10^*B* to the tumor site is needed [1,2,3]. Traditionally, boron-containing molecules, such as boronophenylalanine (BPA), disodium mercaptoundecahydro-*closo*-dodecaborate (BSH), or boranes are infused into the patient, resulting in a tumor-to-normal tissue ratio that, for BPA, is usually taken as 3.5:1 [3,4] The inter-patient variability has been taken into account in some clinical trials, by performing a previous PET scan using BPA labeled with 18F to assess the tumor-to-normal tissue ratio, and a threshold on this value has been set to establish a criterion for patient eligibility [5,6]. Still, developing new and more efficient boron delivery agents is a pressing need. An ideal BNCT agent should selectively deliver large amounts of ^10^*B* to the cancer cell, be eventually detectable by imaging techniques, and present a low clearance [7]. In this respect, nanoparticles of boron-rich inorganic compounds appear particularly promising [8,9,10,11]. For example, a single boron carbide (*B*_4_*C*) nanoparticle of 50 nm in diameter contains, in fact, 7 · 10^6^ atoms of boron, allowing a very localized, high-density *B* delivery. The local buildup of boron atoms achievable through this approach might be enough to avoid using compounds enriched in ^10^*B*, a common practice when borated molecules are used (the natural abundance of ^10^*B* is about 20%). In addition, NPs present a much lower clearance than the easily degradable molecule [10], suggesting the possibility of realizing subsequent BNCT treatments with a single administration. Finally, *B*_4_*C* offers high chemical inertness, ensuring that no harmful components are released during its permanence in the organism [12,13,14,15,16,17].

This work presents the synthesis of polyacrylic acid-stabilized multifunctional nanoparticles based on boron carbide (*B*_4_*C*). To allow imaging of the NP distribution by MRI and optical microscopy, we included these nanoparticles in complex nanostructures, including a gadolinium-containing solid phase and a fluorophore (DiI) [18]. It must be noted that besides allowing MRI imaging, *Gd* compounds contribute to the BNCT activity of the nanoparticles [19]. The isotope ^157^*Gd* has a cross-section for thermal neutron capture of 2.25 · 10^5^ *b*, significantly higher than the one of ^10^*B*. The ^157^*Gd* neutron capture is exothermic, with a *Q*-value of 7.94 MeV, almost all carried by gamma rays [20,21]. Internal conversion (IC) electron release is a competitive mechanism, producing X-rays and Auger electrons. Gamma rays, IC electrons, and Auger electrons are mainly responsible for the biological effects. High-LET electrons are very effective for DNA double-strand breaks. However, because of their very short range in tissues, the ^157^*Gd* atoms must be near the DNA to be effective. On the other hand, gamma rays have a more extended range (>100 μm), so a tumor-killing effect can be achieved even if the nanoparticles are distributed in the proximity of the nucleus and even in the extra-cellular space [22,23].

Preliminary analysis of the interaction of cells with these fluorescent *B*_4_*C* and *Gd*-containing NPs (FGdBNP) was realized using ovary cancer-derived HeLa cell cultures. FGdBNP activity as a BNCT agent was appraised using a correlative microscopy approach combining intracellular neutron autoradiography [24], confocal, and SEM microscopy (correlative light-electron microscopy, CLCM). This new approach allows visualizing the cells, the NPs, and the events deriving from the nuclear process on the same image, confirming the multifunctionality of the FGdBNP.

## 2. Materials and Methods

In this work, we used *B*_4_*C* nanopowder 99% pure from SkySpring Nanomaterials Inc., Houston, TX, USA; polyacrylic acid (PAA, 63% in dH_2_O, MW: 2000 Da) from Polyscience Inc.,Warrington, PA, USA; formaldehyde 16 % from Cell Signaling Technology (Danvers, MA, USA); *Gd*(NO_3_)_3_• H_2_O 99.99% pure from; NH_4_OH (NH_3_ 28–30%) ; DiI (1,1′-Dioctadecyl-3,3,3′,3′-tetramethylindocarbocyanine perchlorate); 3-(4,5-dimethyl thiazolyl-2)-2,5-diphenyltetrazolium bromide; dimethyl sulfoxide; Hoechst 33342; and mowiol (all from Sigma Aldrich, DE St. Louis, MO, USA). dH_2_O was sterilized by filtration with a 0.2 μm pore size filter (Minisart, Sartorious AG, Gottingen, DE).

### 2.1. FGdBNP Syntesis

The composite nanoparticles were obtained starting from commercial *B*_4_*C* nanopowders. These NPs presented a broad particle size distribution and a significant level of agglomeration. To reduce the agglomeration, we first subjected the *B*_4_*C* NPs to a mild ball milling treatment using a planetary mill (*Fritsch pulverisette* 7 *premium line*) with *WC* jars and balls [25]. Various milling treatments have been investigated. The best results were obtained using 2 milling cycles at 200 rpm for 30 min each, with an interval of 5 min between them and a *B*_4_*C*/spheres mass ratio of 1:10. The powders were suspended in 3 mL of water during the milling treatment. 

To improve the stability of the BNP suspension, the NPs were first functionalized with polyacrylic acid (PAA), adapting a protocol optimized previously [26]. Briefly, a suspension of 63 mM BNPs was mixed in a 1:2 ratio with a PAA solution of 1.2% (*w*/*v*). *NH*_4_*OH* 28–30% was slowly added under vigorous stirring to induce PAA complete ionization [27]. A further functionalization with a *Gd*-rich solid phase was obtained by adding to the BNP suspension *Gd*(*NO*_3_)_3_*•* H_2_O in an amount equimolar with *B*_4_*C*. *NH*_4_*OH* 28–30% was slowly added under vigorous stirring, until pH 12. The suspension was maintained at 30 °C under stirring for 48 h and was then centrifuged for 15 min at 1500 rpm (277· *g*, ThermoFisher MicroCL 17, Thermo Fisher Scientific GmbH, Dreierich, DE) to remove the largest aggregates. Next, the supernatant was centrifuged at 1700 rpm (356× *g*) for 15 min. The pellet, representing the fraction of interest, was weighed, resuspended in sterile dH_2_O to obtain a GdBNP concentration of *6* mg/mL, and sonicated for 15 min (*Vevor, MU-*4*L*). The amount of *Gd* in the final suspension is about three times that of boron carbide. A few different B:*Gd* ratios have been investigated. However, the use of ratios different from 1:3 always resulted in unstable nanoparticle suspensions.

To make these GdBNPs fluorescent, we used the solvent diffusion method, proposed by Santra and colleagues [28]. A 1,1′-*Dioctadecyl*-3,3,3′,3′-*tetramethylindocarbocyanine perchlorate* (*DiI*) solution containing 1.2 mg/mL of the chromophore in DMSO was added drop by drop under vigorous stirring to the GdBNP suspension with a volume ratio of 1:20. The stirring was maintained for 45 min. The suspension was centrifuged at 500 g, and the supernatant was discarded to remove the unbound *DiI*. The pellet was resuspended in a volume of dH_2_O equal to the volume of the starting suspension. We evaluated the *DiI* insertion in the GdBNPs by spectrofluorimetric analysis using the plate reader Clariostar (BMG Labtech, Ortenberg, DE).

### 2.2. FGdBNP Characterization

Dynamic light scattering (*DLS*) was performed using a Nano ZS90 DLS analyzer (Malvern Instrument, Malvern, Worcestershire, UK). The suspensions were diluted to about 1 mg/mL for the analysis. We acquired 3 to 5 measures for every sample, each including 14–16 runs, depending on the turbidity. 

X-ray diffraction (*XRD*) analysis was performed using a Bruker D8 Advance diffractometer (Bruker Corp., Billerica, MA, USA) equipped with a copper X-ray source (*λ =* 1.541838 Å) operated at 40 kV and 40 mA. We used the *θ*–*θ*′ configuration, with a step size of 0.05° in 2*θ* and an acquisition time of 10 s for each step. The analysis was performed on the pellet laid on a zero-background sample holder and dried at 80 °C for 1 h.

Micro-Fourier transform infrared spectroscopy (*μ-FTIR*) was realized using a μ-FTIR Nicolet iN10 MX (Thermo Scientific, Dreierich, DE, USA) equipped with a liquid *N_2_*-chilled detector. The analysis was performed in the ATR (attenuated total reflectance) mode in the range 4000–500 cm^−1^ for 22 s. For each sample, 10 μL of the suspension was air-dried on a microscopy glass. Three independent measurements were performed for each sample.

Differential scanning calorimeter-thermo gravimetric analysis (*DSC-TGA*) was realized using an STD Q600 apparatus (TA Instruments, New Castle, Delaware, USA). The analysis was performed in air with a heating rate of 10 °C/min on NPs that were dried in an Abderhalden’s pistol at 50 °C for 1.5 h. In addition, ^1^H-NMR spectra were acquired with the 9.4T Avance III (Bruker Corp, Billerica, MA, USA) in the magic angle spinning (MAS) condition (5–11 Hz) using a 4 μs pulse, 32 scans, and a 5 s delay time. The analysis was performed on dried NPs and pure PAA.

Scanning electron microscopy (*SEM*) and electron-dispersive X-ray spectroscopy (*EDS*) were performed with a TESCAN Mira 3 XMU microscope (TESCAN ORSAY HOLDING s.a., Brno, Czech Republic) equipped with a field emission source and an EDAX microprobe. The microscope was operated between 5 and 25 kV using either SE (secondary electrons) or BSE (backscattered electrons) mode. For the analysis, a drop of nanoparticle suspension was laid directly on a stub and air-dried in a closed container to avoid contamination. Alternatively, a drop of NP suspension was dried on a glass slide using a hot plate. Before the analysis, the sample was carbon-coated (Cressington, Carbon Coater 208 carbon, Cressington Scientific Instruments Ltd., Watford, UK).

For the transmission electron microscopy (*TEM*) analysis, a drop of NP suspension was placed on an ultrathin carbon membrane on a 400-mesh copper grid and left to dry for 5 min. TEM analysis was performed using an FEI TECNAI SPIRIT microscope, working at 80 kV and 300 kV, and equipped with a Bio-Twin lens, *LaB*_6_ electron source, CCD 4 k × 4 k FEI Eagle camera, the Si(Li) EDS, and EDAX detector (Field Electron and Ion Company, Hillsboro, OR, USA). 

The spectrofluorimetric analysis was performed using a plate reader Clariostar (BMG Labtech, Ortenberg, DE). FGdBNP was excited at 472 nm, and the emission spectrum was acquired between 550 and 680 nm with a 1 nm resolution. For each sample, 44 flashpoints were acquired by bottom optics. 

To determine boron content in the GdBNPs, we used a method based on detecting the charged particles emitted by the neutron capture process in ^10^*B* [28]. First, 30 μL of GdBNP suspension was placed directly on a *CR-*39 slide, acting as a passive solid-state nuclear track detector (SSNTD). Then, after evaporation of the solvent, the *CR-*39 was irradiated in the thermal column of the TRIGA reactor of the University of Pavia, with a thermal neutron fluence of about 2 × 10^10^ cm^−2^. The irradiation lasted 30 min at a reactor power of 2 kW. The charged particles emitted by the neutron capture by ^10^*B* produce latent damage in the detector along their path. These tracks become visible after chemical etching, performed with a *PEW*40 solution at 70 °C for 10 min. 

After washing, the tracks were visualized using a Leica MZ16A microscope (Leica, Wetzlar, DE) equipped with lamp Leica CLS150X and joystick PRIOR OPTISCAN II. We used Image Pro Plus 7.0.8 (Media Cybernetics Inc., Rockville, MD, USA) to analyze the images. The measured track density was compared with that obtained by irradiation of a certified sample provided by NIST, constituted by a *Si* wafer with superficially implanted ^10^*B* atoms with a density of 1.018 × 10^15^ atoms cm^−2^. As the nanoparticle layer in the dried samples has a smaller thickness than the charged particles’ penetration range, all the alpha particles produced by the sample can reach the detector (i.e., the dried drop can be considered a superficial distribution of ^10^*B*). Hence, the number of ^10^*B* atoms present in the initial suspension drop can be inferred by comparing the track density obtained by the NIST sample and the dried GdBNP drop of suspension. The ^10^*B* concentration in the original suspension can then be calculated by knowing the volume of the drop. Finally, the results were used to evaluate the nanoparticle administered to cell cultures for intra-cellular boron concentration.

### 2.3. Interaction of GdBNP with Cells

As a model to study the interaction of GdBNPs with biological systems, we used HeLa cells (ATCC^®^, Manassas, VA, USA). Cells were grown in DMEM high glucose medium, added with 10% fetal bovine serum and 2 mM glutamine (all from Euroclone, Pero, Italy), and maintained at 37 °C in a 5% CO_2_ humidified atmosphere. 

The NP toxicity was evaluated by MTT (3-(4,5-dimethyl thiazolyl-2)-2,5-diphenyltetrazolium bromide) assay. Cells were seeded in a 96-well plate (Greiner Bio-One International GmbH, Frickenhausen, DE) at a density of 10,000 cells/well. After 24 h, they were incubated with FGdBNP at 25, 50, 100, and 200 ppm for 30 min, 1 h, 4 h, or 24 h. After washing, cells were grown for 2 h at 37 °C in a fresh medium without nanoparticles but added with MTT 0.5 mg/mL. The medium was then discarded, and, after washing, DMSO was added to allow the dissolution of the formazan crystal. Absorbance at 570 nm was measured by the plate reader. 

For confocal microscopy analysis, HeLa cells were seeded on 18 × 18 mm glass coverslips (Menzel Gläser, Braunschweig, DE). We used cells at about 60% of confluence for incubation with FGdBNPs. For each 35 mm petri dish, 66 μL of FGdBNPs suspension was added. After the treatment, cells were fixed in formaldehyde 4% in PBS for 15 min at room temperature, and nuclei were stained with Hoechst33342. Finally, the coverslip was mounted upside down on a microscope glass slide (Menzel Gläser, Braunschweig, DE) using a drop of *mowiol* mounting agent. For the analysis, we used two different microscopes: a confocal microscope, Leica TCS SP5 II equipped with PL APO 40×/1.25 NA or 63×/1.40 NA objectives (Leica Biosystems, Wetzlar, DE) and motorized stage (defined in the text as CM), and a widefield microscope, Olympus IX83 equipped with 10×, 20× and 60× objectives and a motorized Märzhäuser stage (Olympus Corporation, Hamburg, DE) (defined in the text as OM OPPURE widefield microscope). For FGdBNP visualization, we excited the DiI with a *HeNe* 543 laser and detected the fluorescence using a 579–644 nm passband filter.

For treatment at low temperature, *HeLa* cells were preconditioned for 10 min at 4 °C and then incubated with FGdBNPs for 30 min, maintaining the same temperature. 

For *SEM* analysis, HeLa cells were seeded in a 35 mm plastic petri dish (Corning, Corning, NY, USA). Cells were treated with GdBNPs at about 60% confluency and fixed in cold 70% *Et-OH* at −20 °C for 2 h at the end of the incubation time. After EtOH was discarded, the samples were air-dried. The petri dish was then attached to a SEM stub using conductive double-sided tape, and then the surface was coated with a graphite thin film to make it conductive. 

For the intracellular neutron autoradiography analysis, HeLa cells were seeded directly on a sterilized *CR*39 slide previously marked with three reference points. After 24 h, the cells at 60% confluence were incubated with 50 ppm FGdBNP for 6 h. Cells were then fixed in cold 70% *Et-OH* for 2 h, washed in *PBS*, and images were taken with the Olympus IX83 microscope. Using the motorized stage, we identified the coordinates of a few regions of interest (ROI) selected near the 3 reference points. Next, the sample was irradiated for 2 h with a 250 W neutron flux, according to Postuma et al. [24,29]. To visualize the distribution of the FGdBNPs, the *CR*39 was chemically etched using a 6.25 *N NaOH* solution at 70 °C for 20 min. The *CR*39 slide was then repositioned to the same ROIs defined previously using the corresponding coordinates. At this stage, the cells were no longer visible because the chemical etching destroyed them, but the tracks produced by the α particles could be visualized. For each ROI, the images of the tracks were then overlapped with the corresponding images of the cells to visualize the track distribution. The image overlap was performed using the software *ImageJ (1.46r)*.

To compare the intracellular distribution of the nanoparticles with their BNCT activity, we developed a specific Correlative Light and Electronic Microscopy (CLEM ) analysis. This approach allows observing how the tracks generated on *CR*39 by the neutron capture process correlate with the nanoparticle images obtained by fluorescence and SEM microscopy. For this analysis, *HeLa* cells were seeded on *CR*39 presenting few reference marks and grown as described for the intracellular neutron autoradiography. After fixation, images were acquired using an Olympus IX83 inverted microscope to see the distribution of the fluorescent FGdBNPs within the cells. Subsequently, the *CR*39 was air-dried and treated as described for the SEM analysis, and images of the same ROI defined previously were taken with SEM. The sample was then neutron-irradiated and processed as described for intracellular neutron autoradiography. Finally, the same *CR*39 was analyzed again at the same ROI by optical microscopy to visualize the track distribution generated by the α particles. Finally, the images corresponding to the same ROI acquired with the three different techniques were overlapped using *ImageJ (1.46r)*.

This analysis was also performed on cells treated for 4 h with both BPA (80 ppm) and GdBNP. In the first case, cells were exposed for 4 h to 80 ppm of *BPA*; in the second, for 4 h to 50 ppm of GdBNP. 

## 3. Results and Discussion

### 3.1. Nanoparticles Characterization

**Figure 1:**
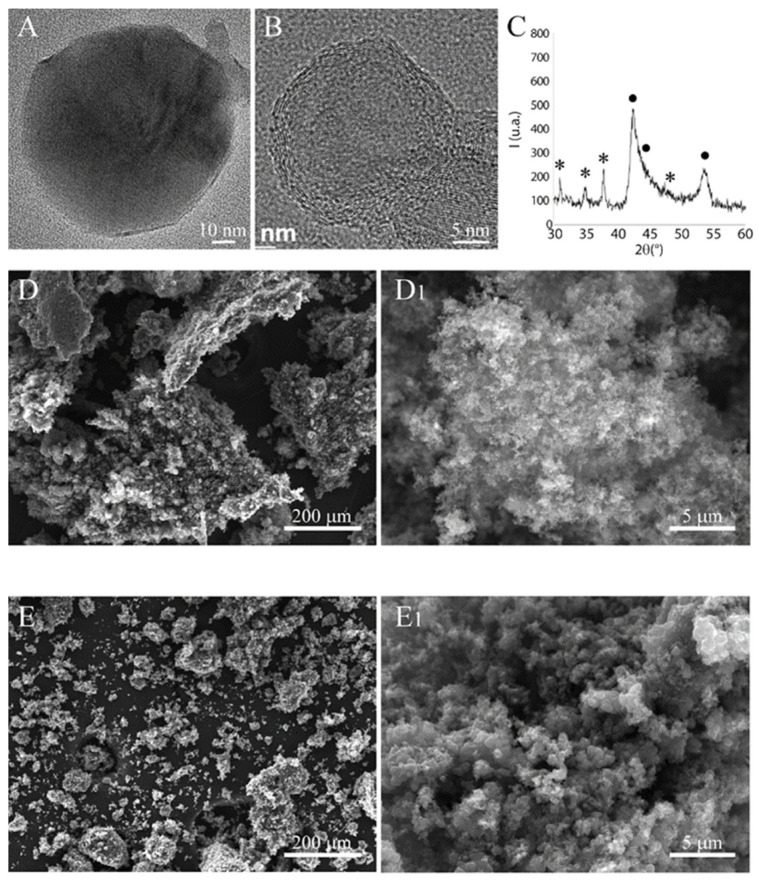
*B*_4_*C* nanopowder characterization. (**A**,**B**) are TEM micrographs acquired at low (80 kV) and high resolution (300 kV), respectively. (**C**) XRD pattern acquired with a step of 0.05° 2θ for 10 s; (•) indicates the graphitic phase while (*) is the boron carbide. SEM micrographs of *B*_4_C NPs before (**D**,**D_1_**) and after (**E**,**E_1_**) ball milling.

We first characterized the starting commercial *B*_4_*C* nanopowders (BNPs) by TEM, SEM, and XRD (Figure 1). TEM images evidenced the presence of spherical nanoparticles with a dimension between 10 and 50 nm. At high resolution (Figure 1B), these BNPs presented a complex structure characterized by a polycrystalline core containing crystallites of *B*_4_*C* of about 1 nm in size, surrounded by a shell composed probably of multiple graphene layers of various thicknesses. The XRD confirms these results (Figure 1C), evidencing two phases: boron carbide and a graphitic fraction. The phase *B*_4_*C* presents broad peaks, indicating the presence of very small crystallites. 

SEM analysis evidenced the extensive agglomeration of the constituent BNPs, forming large aggregates that are several tens of microns in size (Figure 1D,D_1_). The BNPs were subjected to a mild ball milling treatment to reduce this agglomeration. This treatment was only partially successful, leaving a significant level of agglomeration, although reduced in size. A substantial fraction of very small agglomerates could also be observed (see Figure 1E,E_1_).

These BNPs are strongly hydrophobic and cannot be directly used for biological applications. Polyacrylic acid (PAA) was used as a capping agent to improve their stability in the aqueous solution. This functionalization was realized by modifying a protocol originally proposed to synthesize ceria nanoparticles [26]. However, this procedure was only partially successful as the resulting BNPs still presented a significant hydrophobic behavior. The corresponding water suspension resulted in being quite unstable. Dynamic light scattering (DLS) of these PAA-coated BNPs indicated a diameter between 22 and 150 nm. The most significant fraction was removed with mild centrifugation (1700 rpm), resulting in a suspension composed of BNPs with an average size of 52 ± 9 nm. 

To further improve the stability of these BNPs suspension in water, we performed an additional functionalization with *Gd* compounds. Besides increasing the stability of the PAA-BNPs in an aqueous environment, resulting from the inclusion of an ionic compound, this further functionalization offered the possibility of realizing BNPs that MRI could detect. *Gd* compounds are largely used as MRI contrast agents. Furthermore, since *Gd* is a BNCT-active element, it might further enhance the activity of composite nanoparticles upon neutron irradiation. 

The protocol we used for realizing the *Gd*-based functionalization involved the precipitation of a *Gd* oxide/hydroxide in the presence of the BNPs and PAA. With this approach, we produced a suspension of GdBNPs that are stable in water for a few weeks and suitable for biological applications. The successful functionalization of GdBNPs with PAA was confirmed through zeta potential measurements, which recorded a surface charge of −40.8 ± 0.4 mV, ascribable to negatively charged carboxylate groups. 

**Figure 2:**
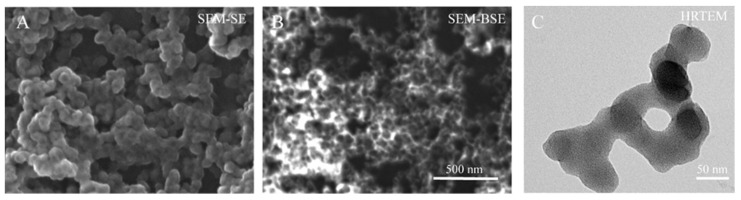
Electron microscopy analysis of GdBNPs. All images refer to the GdBNP fraction obtained after centrifugation and sonication. (**A**,**B**) SEM micrographs of GdBNPs acquired with secondary and backscattered electron detectors, respectively. (**C**) TEM images acquired at 80 kV.

The SEM images of Figure 2 indicate the morphology of these GdBNPs. The SEM image shows particles almost spherical in shape, presenting a homogenous size of about 60 ± 11 nm. On the other hand, the DLS analysis revealed a hydrodynamic diameter of 91 ± 5 nm. Comparing SEM images of the same area obtained using secondary (SE) and backscattered (BS) detectors, it is possible to notice the non-homogeneous distribution of the heavier *Gd* compound within the single nanoparticle. In the BS image, darker round particles are evidenced, probably represented by the lighter *B*_4_*C* phase, surrounded by brighter shells, where the *Gd*-rich compound is located (Figure 2B). The electron dispersive X-ray spectroscopy confirmed the presence of *Gd* (not shown). TEM analysis did not provide further information. Images obtained at 80 kV (Figure 2C) showed the presence of irregular, non-crystalline structures without morphological evidence, which could suggest the presence of *B*_4_*C* cores. However, it must be considered that the amorphous *Gd*-rich matrix might shield the lighter B_4_C core. At 300 kV, the irregular structures resulted unstable, showing evidence of gradual crystallization induced by the exposure to the beam. These results confirm the *B*_4_*C* nanoparticles being surrounded or embedded in an amorphous *Gd*-rich phase.

**Figure 3:**
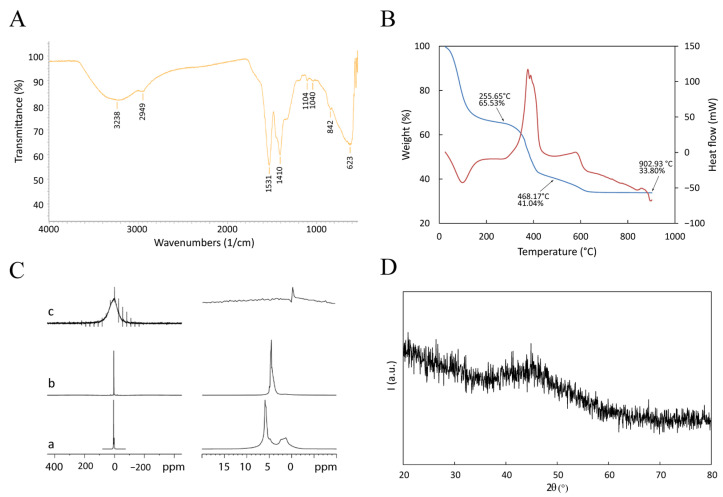
Physical characterization of GdBNP. (**A**) ATR-FTIR spectrum. (**B**) DSC-TGA performed between room temperature and 900 °C. The blue line is the TGA signal; the red line is the DSC signal (Exo up). (**C**) Comparison of ^1^H NMR spectra of PAA solution (**a**), BNP powder (**b**), and GdBNP (**c**). In (**c**), the signals different from that of 0 ppm are artifacts due to the sample rotation (spinning sidebands). Panels on the right show magnifications of the spectral region of the corresponding panels on the left. (**D**) XRD pattern.

μFTIR, DSC-TGA, NMR, and XRD were performed to investigate the chemical nature of the *Gd*-based functionalization (Figure 3).

The μFTIR spectrum (Figure 3A) shows a broad band extending between 3400 and 3200 cm^−1^ due to stretching vibrations of hydroxide groups. Peaks at 1530, 1410, and 1038 cm^−1^ are related to asymmetric stretching, symmetric stretching, and bending of the nitro group within nitrate anions. These ions probably derive from the *Gd* precursor (a nitride). They are included in the GdBNP as either lanthanide-coordinated anions or by intercalation between hydroxide layers to give lanthanide hydroxy nitrates (such as Ln(OH)_2_(NO_3_) • yH_2_O; with Ln = La, Y, *Gd*, etc.) [30]. The broad peak at 628 cm^−1^ is probably related to the bending of -OH groups belonging to a *Gd*-O-H structure. These results suggest that the *Gd* ion could be present in the GdBNP, at least partially, as hydroxy nitrate. The μFTIR spectra do not show any bands ascribable to the carbonyl groups of PAA. This absence could be explained by considering the low amount of PAA compared with the other components.

Thermal analysis of the GdBNP was performed using a combined DSC-TGA apparatus from room temperature up to 900 °C in air. The interpretation of thermograms was made possible by comparison with analogous scans realized on the single components of the GdBNP (see SI materials). Figure 3B shows the presence of three well-defined weight losses. The first, taking place between 25 and 160 °C (about 34.5% of the weight), is associated with an endothermic process and is probably due to the release of water. A second weight loss (24.5%), between 300 and 400 °C, is associated with a complex exothermic process and could probably be ascribed to partial thermal degradation of the PAA. Finally, between 400 and 600 °C, another exothermic process is observed, likely associated with the completion of the thermal degradation of the organic components. No evidence of *Gd* hydroxide or hydroxyl nitrate decomposition can be observed [30].

On the other hand, the NMR analysis suggested the presence of H_2_O or OH groups associated with the *Gd* nuclei (Figure 3C). ^1^H spectra acquired under the MAS condition (5–11 kHz) were compatible with *Gd* hydroxides, even if it was impossible to exclude the presence of *Gd*_3_O_2_. Figure 3C shows the spectra of GdBNPs compared with that of PAA (static spectrum) and PAA-capped BNP powder (9 kHz). While PAA and BNP presented strong signals attributed to mobile protons, the ^1^H spectrum from the GdBNPs sample showed significant paramagnetic enlargement of the peak due to magnetic dipolar interaction. This suggests that in GdBNP, the H_2_O/OH groups are very close to the *Gd* ions.

XRD of the GdBNPs is reported in Figure 3D. It must be noted that there is a complete absence of any clear reflection. Surprisingly, not even the peaks associated with the *B*_4_*C* phase deriving from the starting NPs can be observed (cfr. Figure 1C). This result suggests that the *B*_4_*C* is entirely shielded by the strongly absorbing *Gd*-rich phase that is poorly crystalline.

**Figure 4:**
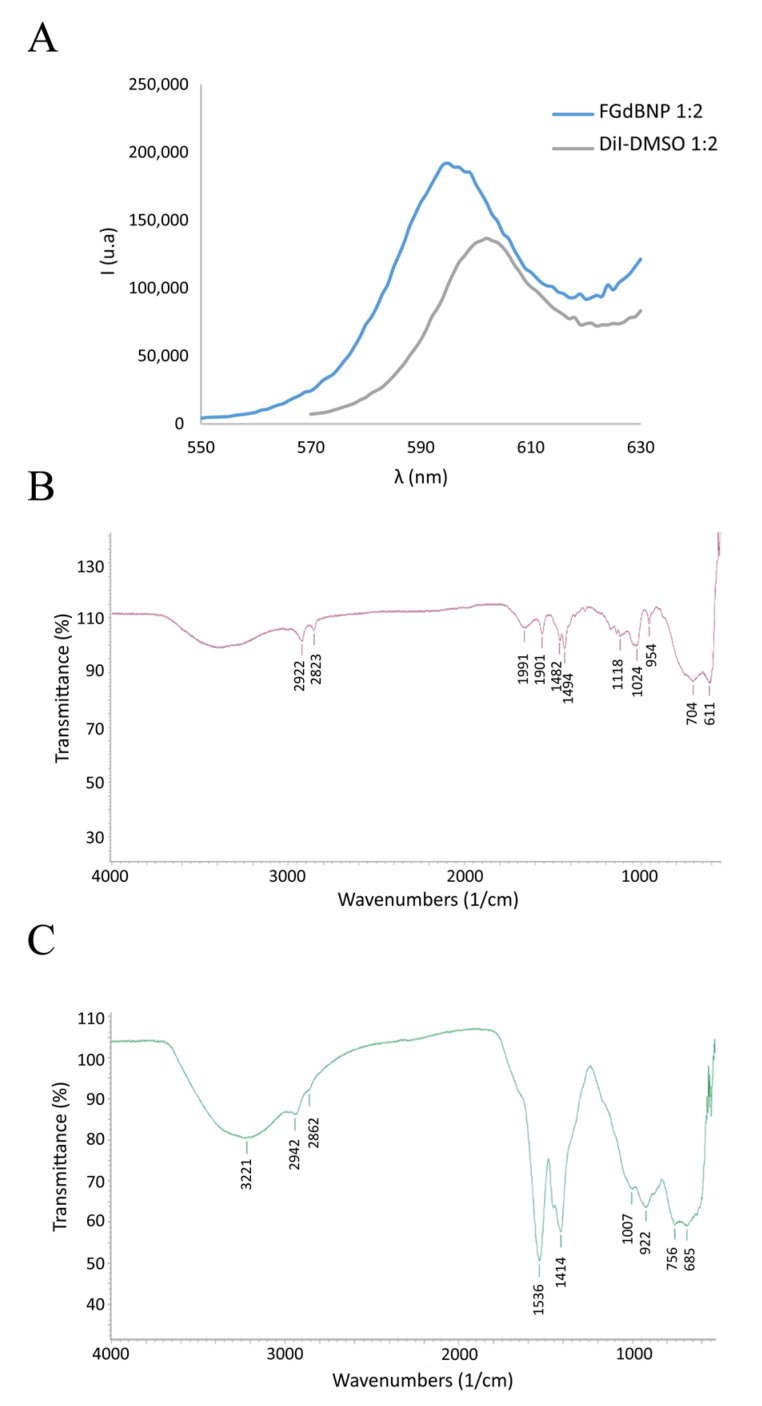
Analysis of fluorescent GdBNP. (**A**) Comparison of emission spectra between DiI in DMSO (gray line) and FGdBNP (blue line). (**B**) ATR-FTIR spectra of DiI. (**C**) ATR-FTIR spectra of FGdBNP.

To make the nanoparticles visible in fluorescence microscopy, the GdBNPs were further functionalized with the fluorescent molecule DiI (1,1′-Dioctadecyl-3,3,3′,3′-tetramethylindocarbocyanine perchlorate), a lipophilic cation that produces a non-covalent hydrophobic interaction with PAA. The optical properties of these NPs have been evaluated by spectrofluorimetric analysis. Figure 4A shows the fluorescence spectra between 550 and 630 nm, resulting from excitation at 472 nm for a suspension of the functionalized GdBNPs compared with that of a solution of DiI in DMSO. A little shift of 10 nm in the maximum of the fluorescence peak can be observed (Figure 4A). This shift has been reported to indicate the successful intercalation of the fluorophore within the PAA hydrophobic microdomains [28].

Further evidence of the successful insertion of DiI into the PAA shell was obtained by μFTIR analysis (Figure 4B,C). The FGdBNP spectrum displayed some additional bands at 2862 cm^−1^, 1007 cm^−1^, and 755 cm^−1^, which are likely related to the presence of the fluorophore. 

### 3.2. Nanoparticle Interactions with Cells

The nanoparticle behavior in biological systems was investigated using HeLa cells. The amount of ^10^*B* present in the GdBNP suspension used for this study was determined by neutron autoradiography and corresponded to a concentration of 0.22 ppm. It must be noted that the nanoparticles were produced with boron in natural isotopic composition. Thus, the total amount of boron in the NP is five times the ^10^*B* concentration evidenced by this technique.

**Figure 5:**
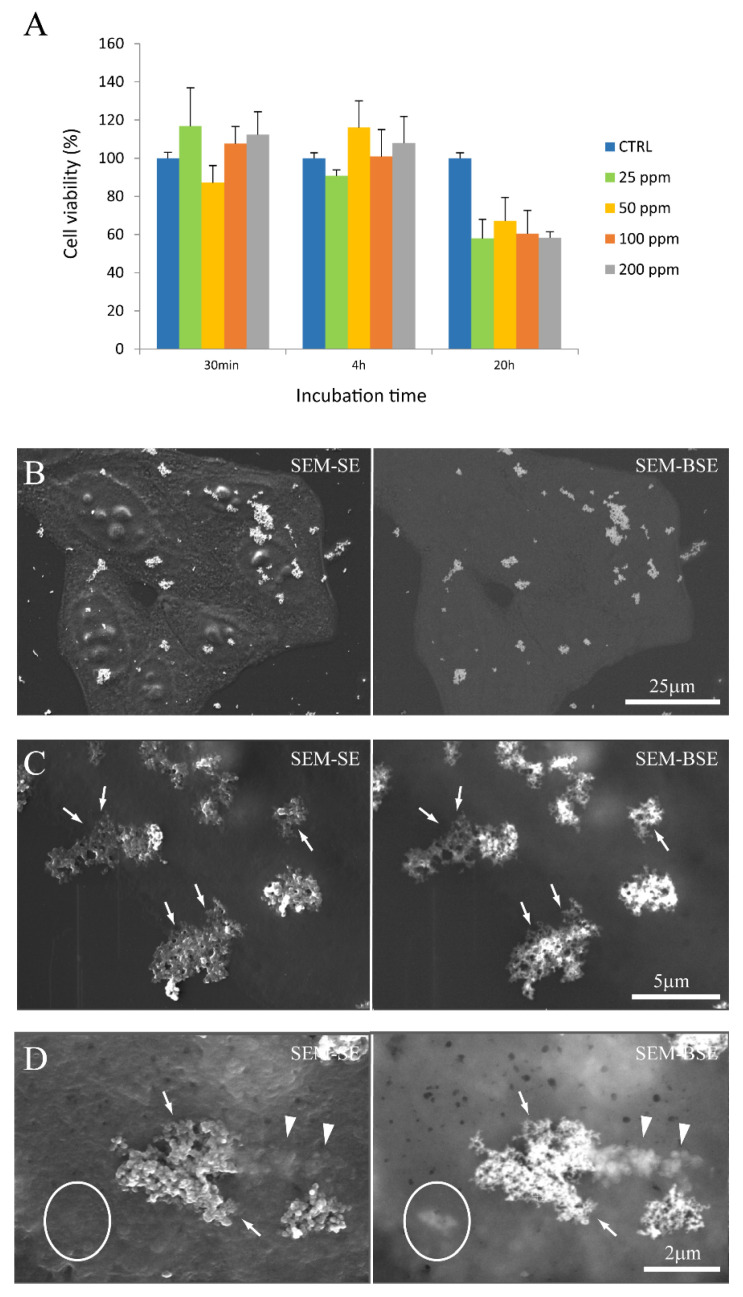
GdBNPs–cell interaction. (**A**) Analysis of GdBNP toxicity by MTT assay. HeLa cells were incubated with different concentrations of GdBNPs for different incubation times. Control is fixed at 100%. Each value is the mean ± DS of three independent experiments. (**B**–**D**) SEM analysis of GdBNPs–cell interaction. (**B**) GdBNPs (50 ppm) incubated with HeLa cells for 30 min are visible as aggregates (white spots) on a group of cells. (**C**,**D**) GdBNPs interacting with the cell membrane. (**C**) NP agglomerates adherent to the cell surface with their edges partially underneath the cell membrane (arrows). (**D**) GdBNPs internalization process at an early stage. The aggregate is partially outside, looking bright and well on-focus (arrows), and partially underneath the membrane, as it appears blurred (arrowheads). Aggregates entirely captured by the cell are completely out-of-focus (encircled). Image (**B**) was acquired at 5 kV; images (**C**,**D**) were acquired at 15 kV of acceleration voltage.

We first evaluated the NP cytotoxicity by MTT assay. Figure 5A shows the viability of cells after incubation with FGdBNP at different concentrations and at different times. Each value is the mean of three independent experiments and is expressed as % of viability with respect to control cells (absence of FGdBNPs). The histograms of Figure 5A show that FGdBNP toxicity can be observed only for the highest concentration (200 ppm) or for the most extended incubation times (24 h). Based on these results, we decided to perform the subsequent characterizations using GdBNPs at a concentration of 50 ppm for incubation times of 6 h.

The interaction of FGbBNPs with cells has been investigated by electron microscopy (SEM) and fluorescence confocal microscopy. In the SEM images, the GdBNPs appear as brighter spots due to the high atomic weight of *Gd* (Figure 5B–D). This contrast was enhanced using a backscattered detector (BSE). In these images, the GbBNPs can mainly be observed in large aggregates present on the cell membrane or just underneath it (Figure 5B–D). The adhesion of the GdBNPs to the cell membrane was evident at higher magnification (Figure 5C,D). Here, some aggregates appear to be strongly connected or partially embedded under the cell membrane and appear almost “fused” with the biological structures (Figure 5D). The NPs internalization, at its early stage, is also evidenced in these images. In some instances (Figure 5D), part of the aggregate appears to be still on the outside of the membrane, appearing very bright and on focus, while another portion, appearing blurred and out of focus, has already crossed the membrane (Figure 5D). Aggregates of GdBNPs that are already entirely underneath the cell membrane would not be seen in SE but only as blurred spots in BSE (Figure 5D, circled). These observations suggested that, despite the aggregation and the *Gd* functionalization, the GdBNPs can easily be internalized.

MTT assay showed no significant cytotoxicity for ^10^*B* concentrations up to 100 ppm and 4 h incubation time. The nature of the limited toxicity of the FGdBNPs was not investigated further. The main concern regarding using *Gd*-based contrast agents is linked to the development of nephrosclerosis. However, nephrosclerosis is a medical complication reported in relation to continuous administration for long periods of exposure to *Gd*-based contrast agents (GBCAs) for MRI. It must be stressed that *Gd* in GdBNPs is not available in solution as a complexed ion but as a solid phase. Although it might be possible that the solid phase might release some *Gd* ions, the MTT assay did not show any evidence of acute cytotoxicity at the cellular level. 

**Figure 6:**
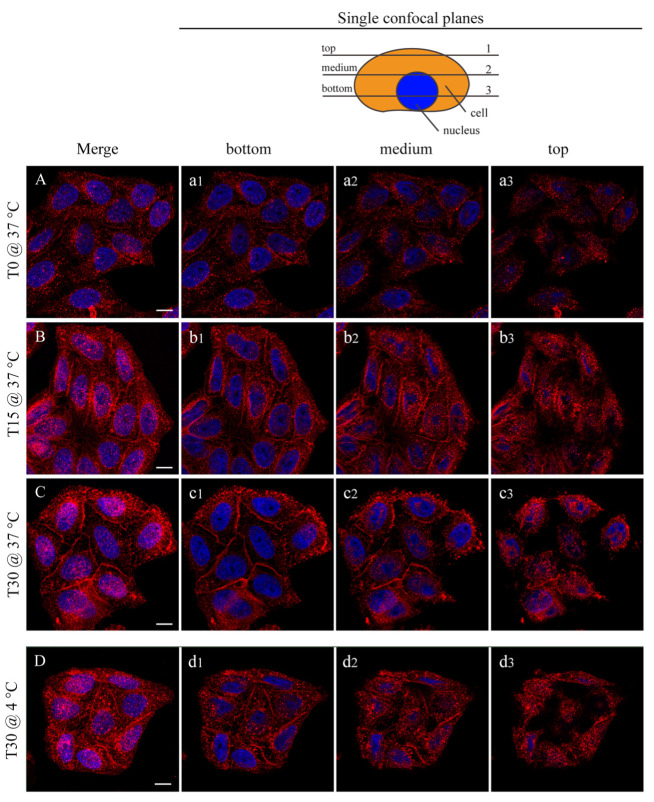
Confocal microscopy analysis of FGdBNP–HeLa cells interaction at different incubation times and temperatures. HeLa cells were incubated with 50 ppm FGdBNP at 37 °C for 0 min (NPs were added and immediately removed ((**A**), (**a_1_**–**a_3_**)), 15 min ((**B**), (**b_1_**–**b_3_**)), and 30 min ((**C**), (**c_1_**–**c_3_**)). Images (**A**–**C**) are Z projections of all the acquisition planes. (**a_1_**–**a_3_**), (**b_1_**–**b_3_**), and (**c_1_**–**c_3_**) are single z-planes (see schematic in the upper right corner). (**D**) HeLa cells incubated with FGdBNP at 4 °C per 30 min (Z projection). (**d_1_**–**d_3_**) are single Z-planes. Red, FGdBNPs. Blue, cell nuclei. Bar is 10 μm.

In Figure 6, the interaction between the HeLa cells and the NP is shown by confocal microscopy. In this case, the fluorescent FGdBNPs have been used. Three incubation times are shown: T0, where the NPs addition was followed by an immediate removal, 15 min, and 30 min. By comparing Figure 6A–C, it can be noted that there is an increase in FGdBNPs both adherent to the cell membrane and being internalized, as the overall fluorescence increases over time. The red spots, due to the FGdBNPs attached or internalized by the cells, increase with the incubation time.

Surprisingly, the interaction of the FGdBNPs with the cells was rapid, as red spots were already visible on all cells, even if the contact time had been extremely short (Figure 6A,a_1_–a_3_). In this case (T0), the medium containing the FGdBNPs was discharged almost instantly after the beginning of the incubation. Furthermore, this rapid interaction was also observed using cells whose active biological processes were prevented or inhibited by maintaining them at 4 °C during the incubation with FGdBNPs. Cellular endocytosis is almost completely blocked at this temperature, and only passive binding mechanisms remain active. Figure 6D shows HeLa incubated with FGdBNPs for 30 min at 4 °C. They show significant uptake of NPs, comparable to that observed with cells incubated at 37 °C (Figure 6D vs. Figure 6C). This observation indicates the existence of a fast chemical interaction between the FGdBNPs and the cell surface, which differs from the energy-dependent internalization processes. 

**Figure 7:**
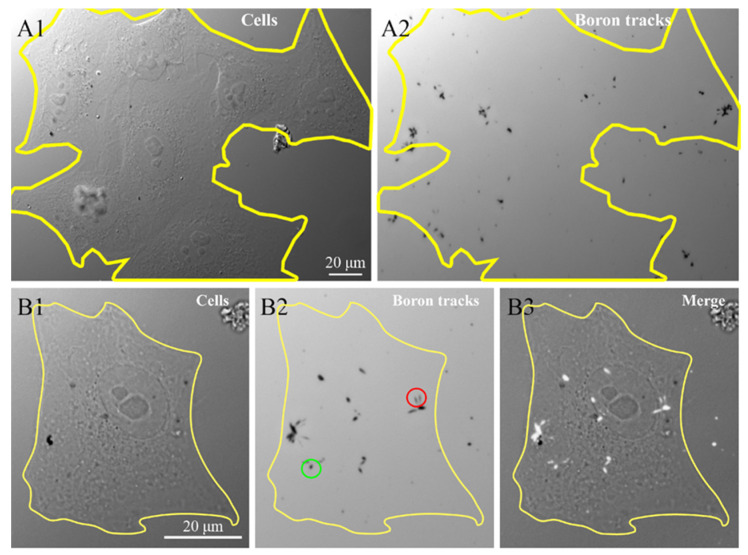
Intracellular neutron autoradiography. HeLa cells grown on CR39 were treated for 6 h with 50 ppm GdBNP, fixed, and then subjected to neutron irradiation. (**A1**) image of cells acquired before neutron irradiation. (**A2**) traces (black marks) produced by the α-particle on CR39 after irradiation. The yellow line in (**A1**) outlines a cluster of about seven cells, which is no longer visible in (**A2**) after track development. (**B1**,**B2**) a single cell before and after the track’s development. The green circle in (**B2**) outlines an example of a circular track derived from a perpendicular α-particle trajectory. In contrast, the red circle indicates two oblong marks produced by oblique α-particles. (**B3**) is the overlap between (**B1**) and (**B2**), where the α tracks are evidenced as white signs overlapped on the original cell image.

To confirm the presence of ^10^*B* containing NP in the cells, we investigated the ^10^*B* distribution at the intra-cellular level using the neutron autoradiography procedure described by Postuma et al. [24]. This procedure helps to clarify the correlations between the microscopic localization of the GdBNPs and the potential damage resulting from the neutron capture. Figure 7 shows the images, taken with the widefield microscope, of cells grown and exposed for 6 h with 50 ppm GdBNPs on the CR39 detector before irradiation (Figure 7(A1,B1)) and the tracks produced in the same region of the CR39 from the α particles after the neutron irradiation process, visible as black marks (Figure 7(A2,B2)). Figure 7B3 shows the overlap between the images relative to the cell before irradiation and the charged particle tracks produced by the irradiation (Figure 7(B3)). This analysis confirms the intracellular localization of ^10^*B*, which has been internalized as GdBNP. It must be noted that the different shape of the tracks, circular or oblong (green and red circles, respectively, in Figure 7), gives indications of the direction of the α-particle when they crossed the CR39, thus on the original position of boron atoms within the cell. 

**Figure 8:**
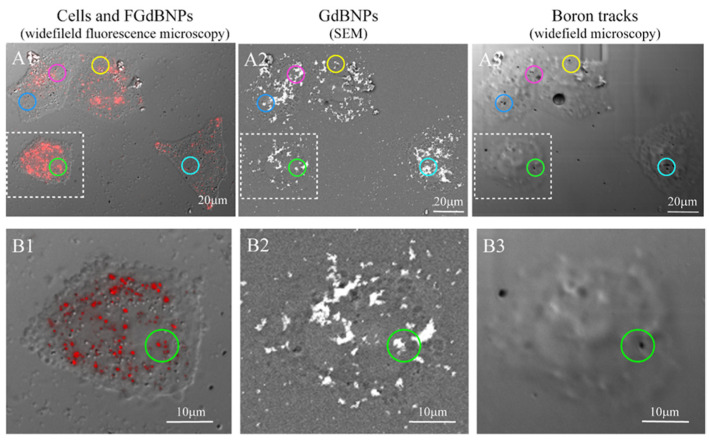
Correlative light and electron microscopy (CLEM) analysis of the distribution of the FGdBNPs. HeLa cells were grown on CR39 and treated for 6 h with 50 ppm GdBNPs. Example of the same area analyzed first by widefield fluorescence microscopy (**A1**,**B1**), then by SEM-BSE (**A2**,**B2**), and again by widefield microscopy after neutron irradiation and track development (**A3**,**B3**). The colored circles evidence a few examples of FGdBNPs visible as a red spot in confocal microscopy (**A1**,**B1**), white mark in SEM-BSE (**A2**,**B2**), and black tracks on the CR39 (**A3**,**B3**). In (**A3**,**B3**), cells imprinting are visible, although cells were not present anymore. This effect was due to the damage produced on CR39 by the electron beam during the SEM analysis.

**Figure 9:**
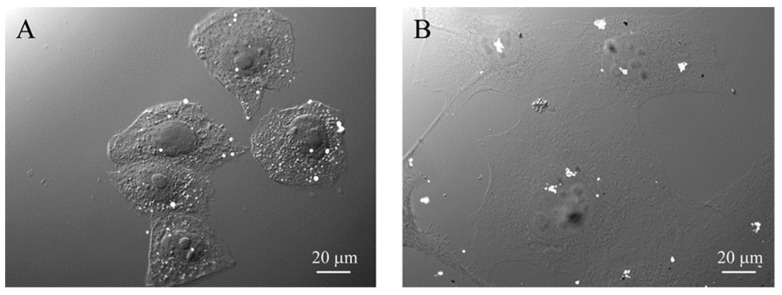
Comparison of the microscopic distribution of α-particle tracks (white dots) on CR39 generated by *^10^B* in cells treated with (**A**) BPA (80 ppm) or (**B**) GdBNPs (50 ppm).

We extended this approach further, performing the SEM-BSE and fluorescence microscopy characterization on the same cells. This correlative light and electron microscopy (CLEM) analysis allowed obtaining an unprecedented characterization of the morphological and functional results of the cell incorporation of GdBNPs. For this CLEM analysis, the cells were first analyzed by fluorescence optical microscopy to detect the distribution of the NPs. Then, the same area was analyzed by SEM-BSE, and subsequently exposed to neutron irradiation. The resulting α-particles tracks in the same regions were imaged again by optical microscopy. Figure 8 summarizes all the stages of this correlative analysis. The circled areas in Figure 8(C1–C3) evidence regions where it can be easily noted the correspondence between the FGdBNPs, which appears as a red spot in light microscopy (Figure 8(C1)) and as a white mark in SEM-BSE (Figure 8(C2)), and the tracks produced by the α-particles, appearing as a black spot, on the CR39 (Figure 8(C3)). It must be noted that the non-perfect correspondence between the GdBNPs distribution observed in the same areas by fluorescence microscopy and SEM (Figure 9(A1) vs. Figure 9(A2,B1) vs. Figure 9(B2)) is caused by some level of cell deformation produced by the complex technical procedure. Cells observed after fixation by confocal microscopy must be dehydrated and carbon-coated to be analyzed by SEM analyses. These procedures might cause a shift or loss of some intracellular structures. It is also important to point out that not all the GdBNPs visible by fluorescence microscope and SEM produce tracks on CR39 after neutron irradiation (Figure 8(A1,A2) vs. Figure 8(A3) and Figure 8(B1,B2) vs. Figure 8(B3)). This is due to the statistics of the irradiation process, which is controlled by the nuclear cross-section and neutron fluence, that in our study has been optimized to obtain separate tracks and allow proper imaging.

The micro localization of boron due to the GdBNP was finally compared with that of Boronophenylalanine (BPA), the standard drug used in clinical BNCT. Figure 9A shows track distributions in CR-39 for cells exposed to BPA, while Figure 9B shows the results due to GdBNP. In this image, the tracks are visible as white dots. These images show that the number of tracks in the cells is comparable, the main difference being the distribution of the tracks. Tracks from BPA appear to be isolated, while tracks from GdBNP are grouped in clusters. It must be noted that while the BPA was isotopically enriched with ^10^*B*, the nanoparticles contained ^10^*B* in amounts corresponding to its natural abundance.

## 4. Conclusions

We explored the possibility of using boron-containing nanoparticles based on low-cost, commercial *B*_4_*C* as a vector for boron delivery in BNCT. The NPs have been functionalized using simple and inexpensive approaches to make them biocompatible and detectable by different imaging techniques. Despite a significant level of agglomeration, the composite nanoparticles were easily and rapidly incorporated by the cells. The resulting toxicity levels have been relatively low and compatible with their use as boron carriers. 

The co-localization technique we developed for this study, combining optical and SEM microscopy with neutron autoradiography, allowed us to characterize the composite nanostructures’ multiple functionalities at the cellular level. It confirmed that none of the components included in the nanostructures, the boron carbide core, the *Gd*-rich solid phase, and the fluorophore, are lost when the nanostructures are placed in the culture medium during their cell incorporation and compartmentation. This conclusion is particularly relevant, considering that the interaction between the various components does not involve the formation of strong chemical bonding. The observed low toxicity level also excludes the possibility of any relevant intracellular release of toxic *Gd* compounds deriving from the partial dissolution of the *Gd*-rich solid phase.

Although the ability of these nanostructures to selectively accumulate in tumoral tissues is still to be proved, it is interesting to note that the distribution of the α-particle tracks produced at the cellular level appears to be qualitatively comparable with that observed with isotopically enriched BPA. This observation represents a promising result given the possible use of these nanostructures in BNCT treatment. 

Further functionalization will be required to achieve selective targeting of specific tumoral structures. This will be the object of a subsequent study. 

Combining boron and gadolinium in the same formulation requires the evaluation of possible synergistic effects that could, in turn, demand further optimization of the ratio between ^10^*B* and ^157^*Gd*. The presence of *Gd* could cause a shielding effect and thus reduce the effectiveness of neutron irradiation [31]. Moreover, dosimetry in the presence of *Gd* is particularly challenging due to the broad spectrum of secondary radiation and the biological effect linked to the capacity of *Gd* to reach the cell nucleus. For this reason, the imaging of nanoparticles at a subcellular level will play an important role. This further optimization of the nanoparticles will be possible when the in vivo phase of the research is designed and carried out.
